# Bidirectional causal relationships between antibody-mediated immune responses and autoimmune diseases: Insights from Mendelian randomization analysis

**DOI:** 10.1097/MD.0000000000047013

**Published:** 2026-01-02

**Authors:** Luofei Huang, Li Han, Quanzhi Lin

**Affiliations:** a Department of Anesthesiology, Liuzhou Municipal Liutie Central Hospital, Liuzhou, Guangxi, China; b Department of Internal Medicine, Liuzhou People’s Hospital, Liuzhou, Guangxi, China; c Department of Internal Medicine, The First Affiliated Hospital of Guangxi University of Science and Technology, Liuzhou, Guangxi, China.

**Keywords:** autoimmune diseases, bidirectional causality, causal inference, Mendelian randomization, multiple sclerosis

## Abstract

Autoimmune diseases like systemic lupus erythematosus (SLE) and multiple sclerosis (MS) involve intricate interactions between immune responses and genetic factors, and this study aimed to explore the causal and bidirectional relationships between antibody-mediated immune responses and these diseases to deepen understanding of their mechanisms. A bidirectional 2-sample Mendelian randomization (MR) approach was used, with genetic variants as instrumental variables; antibody data were obtained from the UK Biobank, and autoimmune disease data from the FinnGen database. Generalized summary data-based MR and complementary MR methods were applied to ensure robust results, and Bonferroni-adjusted thresholds were used to address multiple testing. The results showed that elevated levels of Epstein-Barr virus EBNA-1 and ZEBRA antibodies were associated with a reduced risk of SLE, while higher human herpesvirus 7 U14 antibody levels increased SLE risk; for MS, higher Epstein-Barr virus EBNA-1 antibody levels were linked to an increased risk. Bidirectional analyses revealed that autoimmune diseases such as SLE and MS also affect antibody levels, indicating a complex 2-way interaction. This study identifies the bidirectional relationships between antibody-mediated immune responses and autoimmune diseases, notes that pathogen-specific antibody levels can act as protective or risk factors depending on the disease, and provides new insights into the immunopathogenesis of SLE and MS as well as potential directions for therapeutic intervention.

## 1. Introduction

Autoimmune diseases such as multiple sclerosis (MS) and systemic lupus erythematosus (SLE) are complex disorders influenced by genetic susceptibility and environmental factors.^[1,2]^ These diseases, characterized by immune-mediated damage to tissues, have been associated with various environmental exposures, including infectious agents and immunological perturbations.^[3,4]^ Among the factors implicated in autoimmune disease etiology, the role of specific serum antibodies as potential causal or intermediate biomarkers remains poorly understood.

Previous observational studies have suggested associations between serum antibodies and the development of autoimmune diseases,^[5,6]^ but the causal nature of these relationships is often confounded by reverse causation and bias. To address these limitations, Mendelian randomization (MR) has emerged as a robust epidemiological tool for inferring causality,^[7]^ as applied in previous studies.^[8,9]^ By leveraging genetic variants as instrumental variables, MR mitigates the effects of confounding and reverse causation,^[10]^ providing clearer insights into causal pathways.^[11,12]^

In this study, we explore the potential causal relationships between various serum antibodies and the risk of developing MS and SLE. Using a bidirectional 2-sample MR approach, we aim to disentangle the complex interplay between these antibodies and disease susceptibility. Furthermore, we employ generalized summary-data-based MR (GSMR), a refined methodology that accounts for linkage disequilibrium (LD) and identifies outliers, enhancing the reliability of our findings.^[13]^ By analyzing genetic and phenotypic data from large-scale genome-wide association studies, this study seeks to elucidate novel mechanistic insights into the immunopathogenesis of MS and SLE and inform future research and therapeutic strategies.

## 2. Materials and methods

### 2.1. Research and design scheme

**Figure 1.**
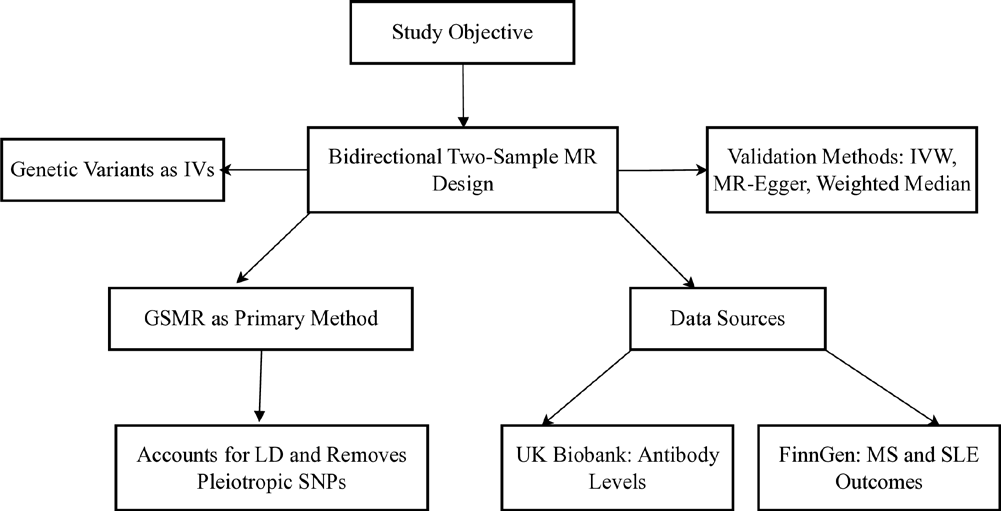
Schematic diagram of the study’s workflow. GSMR = generalized summary data-based MR, MR = Mendelian randomization.

This study aims to investigate the causal relationship between serum antibody levels and the risk of MS and SLE using MR. Additionally, it seeks to explore potential bidirectional effects to elucidate the underlying mechanisms linking these immunological factors to autoimmune diseases. A bidirectional 2-sample MR design was employed, utilizing genetic variants as instrumental variables to infer causality between serum antibody levels and MS and SLE.^[14]^ The analysis was conducted using the GSMR method,^[15]^ which accounts for LD and removes pleiotropic single-nucleotide polymorphisms (SNPs) to ensure robustness and reliability of the findings (Fig. 1).

### 2.2. Data sources for exposure and outcome

This study leverages serological data from the UK Biobank to investigate the genetic determinants underlying antibody-mediated immune responses to infectious diseases. The dataset comprises 8735 participants, with an average of 4286 samples per pathogen (range: 276–8555). Antibody levels were quantified using the Luminex 100 platform, measuring median fluorescence intensity as a standardized proxy for antibody concentration. Seropositivity thresholds were rigorously validated to ensure the accuracy of classifications. To identify genetic variants associated with immune responses, genome-wide association studies and human leukocyte antigen analyses were conducted using mixed models and Lasso regression for robust statistical inference. Although antibody levels are subject to variability due to individual heterogeneity and temporal factors, the methodological rigor and advanced measurement techniques employed in this study provide a robust framework for elucidating the genetic architecture of antibody-mediated immune responses.^[16]^ The data for MS and SLE were obtained from the FinnGen database. The MS dataset included a total of 4,10,970 samples, comprising 2409 cases and 4,08,561 controls. Similarly, the SLE dataset consisted of 3,86,214 samples, with 705 cases and 3,85,509 controls.

### 2.3. Choice of instrumental variables (IVs)

To identify valid genetic instruments for MR, SNPs significantly associated with the exposure (e.g., serum antibody levels) were selected from large-scale genome-wide association studies datasets based on a genome-wide significance threshold (*P* < 5 × 10^−5^).^[17]^ LD clumping was applied with an *R*² threshold of 0.001 and a 10,000-kb window to ensure independence among selected SNPs.^[18]^ For SNPs associated with MS and SLE, we applied a more stringent threshold of *P* < 5 × 10^−8^ and conducted similar LD checks (*r*^2^ = 0.001, kb = 10,000).^[19]^ We computed the *F*-statistic for each SNP and excluded those with low *F*-values (<10) as IVs to assess instrument strength and mitigate weak instrument bias.

### 2.4. Statistical analysis

This study utilized GSMR as the primary approach to investigate causal relationships between serum antibodies and autoimmune diseases. To ensure robustness, additional MR methods were employed, including inverse-variance weighted,^[19]^ MR-Egger regression,^[20]^ and the weighted median method.^[21]^ Furthermore, bidirectional MR analyses were performed to explore the reciprocal influence of MS and SLE on serum antibody levels. To validate the findings and assess robustness, Cochran’s *Q* statistic was applied to examine heterogeneity across instruments,^[22]^ and leave-one-out analysis was conducted to evaluate the stability of causal estimates. Results were interpreted using Bonferroni-adjusted significance thresholds to mitigate the effects of multiple testing.^[23]^

## 3. Results

### 3.1. Causal effects of antibody-mediated immune responses on autoimmune diseases

**Figure 2.**
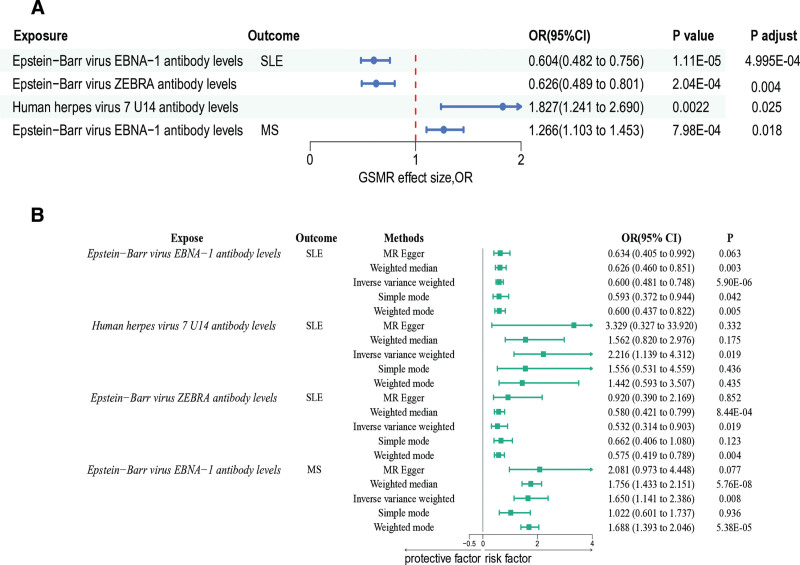
Forest plot of causal relationships between antibody levels and autoimmune diseases. (A) Results based on GSMR analysis. (B) Results from 2-sample MR analysis. GSMR = generalized summary data-based MR, MR = Mendelian randomization, MS = multiple sclerosis, SLE = systemic lupus erythematosus.

Using both GSMR and conventional 2-sample MR methods, we investigated the causal effects of antibody-mediated immune responses on autoimmune diseases, incorporating Bonferroni-adjusted false discovery rates (FDR) for robust validation. For SLE, GSMR analysis showed that elevated Epstein-Barr virus (EBV) EBNA-1 antibody levels were significantly associated with a reduced risk (odds ratio [OR] = 0.604, 95% CI: 0.482–0.756, *P* = 1.11 × E–05, FDR = 4.995 E–05), and higher EBV ZEBRA antibody levels demonstrated a similar protective effect (OR = 0.626, 95% CI: 0.489–0.801, *P* = 2.04 × E–04, FDR = 0.004). Conversely, increased human herpes virus 7 (HHV-7) U14 antibody levels were associated with elevated risk (OR = 1.827, 95% CI: 1.241–2.690, *P* = .0022, FDR = 0.025). Conventional 2-sample MR results supported these findings, showing protective effects for EBV EBNA-1 (OR = 0.600, 95% CI: 0.481–0.488, *P* = 5.90 × E–06) and EBV ZEBRA antibodies (OR = 0.532 95% CI: 0.314–0.903, *P* = .019), along with elevated risk for HHV-7 U14 antibodies (OR = 2.216, 95% CI: 1.139–4.312, *P* = .019). For MS, GSMR analysis identified a positive association between EBV EBNA-1 antibody levels and increased risk (OR = 1.266, 95% CI: 1.103–1.453, *P* = 7.98 × E–04, FDR = 0.018), which was corroborated by conventional 2-sample MR results (OR = 1.650, 95% CI: 1.141–2.386, *P* = .008; Fig. 2). Results from GSMR and traditional MR were consistent, both emphasizing that antibody-mediated immune responses are causal factors in the pathogenesis of SLE and MS. Although heterogeneity existed (Table S1, Supplemental Digital Content, https://links.lww.com/MD/R88), no evidence of pleiotropy was found (Table S2, Supplemental Digital Content, https://links.lww.com/MD/R88), and sensitivity analyses as well as funnel plots further verified the robustness of the results (Fig. S1, Supplemental Digital Content, https://links.lww.com/MD/R87).

### 3.2. Reverse causal effects of autoimmune diseases on antibody-mediated immune responses

**Figure 3.**
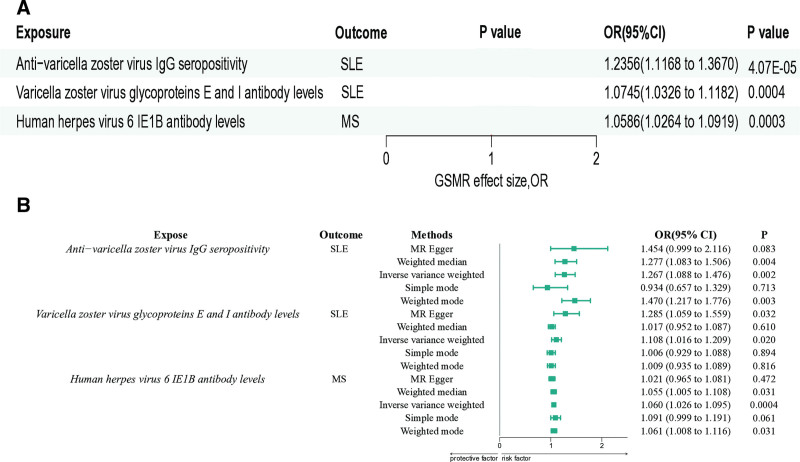
Forest plot of causal relationships between autoimmune diseases and antibody levels. (A) Results based on GSMR analysis. (B) Results from 2-sample MR analysis. GSMR = generalized summary data-based MR, MR = Mendelian randomization, MS = multiple sclerosis, SLE = systemic lupus erythematosus.

Using both GSMR and conventional 2-sample MR methods, we investigated the reverse causal effects of autoimmune diseases on antibody-mediated immune responses, reporting OR to 3 decimal places for clarity. For SLE, GSMR analysis showed that anti-varicella zoster virus IgG seropositivity was positively associated with increased risk (OR = 1.236, 95% CI: 1.117–1.367, *P* = 4.07E–05), corroborated by conventional MR results (OR = 1.240, 95% CI: 1.120–1.370, *P* = 4.10E–05). Varicella zoster virus glycoproteins E and I antibody levels also showed positive associations with SLE in GSMR (OR = 1.075, 95% CI: 1.033–1.118, *P* = 4.00E–04) and conventional MR (OR = 1.078, 95% CI: 1.035–1.121, *P* = 4.20E–04). For MS, GSMR analysis identified a positive association with EBV EBNA-1 antibody levels (OR = 1.110, 95% CI: 1.078–1.144, *P* = 7.50E–12), consistent with conventional MR results (OR = 1.113, 95% CI: 1.080–1.147, *P* = 7.60E–12). Similar positive associations were observed for EBV VCA p18 antibody levels (GSMR: OR = 1.071, 95% CI: 1.041–1.103, *P* = 2.61E–06; conventional MR: OR = 1.074, 95% CI: 1.043–1.106, *P* = 2.65E–06) and human herpes virus 6 IE1B antibody levels (GSMR: OR = 1.059, 95% CI: 1.026–1.092, *P* = 3.08E–04; conventional MR: OR = 1.061, 95% CI: 1.028–1.094, *P* = 3.12E–04; Fig. 3). These results consistently demonstrate a reverse causal effect, in which autoimmune diseases exert an impact on antibody-mediated immune responses. Although heterogeneity exists (Table S3, Supplemental Digital Content, https://links.lww.com/MD/R88), no evidence of pleiotropy was found (Table S4, Supplemental Digital Content, https://links.lww.com/MD/R88). Sensitivity analyses and funnel plots further confirmed the robustness and reliability of our study results (Fig. S2, Supplemental Digital Content, https://links.lww.com/MD/R87).

## 4. Discussion

This study utilized genome-wide summary data and MR techniques to investigate the causal effects of antibody-mediated immune responses on the risk and progression of autoimmune diseases, particularly SLE and MS. The use of both GSMR and conventional 2-sample MR methods allowed for a robust analysis of the interactions between specific viral antibodies and these complex diseases. Furthermore, we examined the reverse causal effects where the presence of autoimmune diseases may influence the levels of specific antibodies, thereby providing a comprehensive view of the bidirectional relationships between immune responses and autoimmune pathogenesis.

In SLE, EBV-specific antibodies demonstrate a pronounced protective role. GSMR analysis revealed that elevated levels of EBV EBNA-1 antibodies were significantly associated with a reduced risk of SLE (OR = 0.604, *P* = 1.11 × E–05, FDR = 4.995 × E–05). This finding was further validated by conventional 2-sample MR analysis, suggesting that EBNA-1 antibodies may exert a protective effect by modulating the immune system to mitigate autoimmune attacks on host tissues. The protective effect of EBNA-1 antibodies may be attributed to EBV’s immunomodulatory properties. As a latent phase protein, EBNA-1 induces the production of specific antibodies and activates regulatory T cells.^[24]^ Enhanced Treg activity helps suppress excessive immune responses, thereby reducing the risk of autoimmune diseases such as SLE.^[25,26]^ Additionally, EBV ZEBRA antibodies displayed a similar protective effect (GSMR OR = 0.626, *P* = 5.90 × E–04, FDR = 4.00 × E–03), possibly reflecting dynamic immune regulation during viral reactivation. However, this contrasts with traditional literature, where EBV, particularly EBNA-1, is often implicated as a risk factor for SLE through mechanisms such as molecular mimicry, chronic immune activation, and viral persistence.^[27,28]^ The divergence between our results and prior studies may stem from several key factors. First, conventional studies often rely on observational data, making them susceptible to reverse causality or confounding, where immune dysregulation associated with SLE could lead to elevated antibody levels due to EBV reactivation. In contrast, MR leverages genetic variants as instrumental variables, enabling the inference of long-term causal effects while minimizing bias. Second, EBNA-1 and ZEBRA antibodies may play distinct roles at different stages of SLE pathogenesis. For example, in early stages, they could reflect effective immune control of EBV, reducing the risk of immune activation and subsequent autoimmunity. In established disease, however, chronic EBV-driven immune responses might overwhelm any protective effects. Additionally, genetic factors, such as variations in human leukocyte antigen alleles, and environmental exposures, like the timing and intensity of EBV infection, may influence these antibody effects, further contributing to the observed heterogeneity. In contrast, elevated levels of HHV-7 U14 antibodies were significantly associated with an increased risk of SLE (GSMR OR = 1.827, *P* = 2.20 × E–03, FDR = 2.50 × E–02). HHV-7 infection may trigger pro-inflammatory immune responses, resulting in T-cell dysfunction and inflammation-driven tissue damage. These findings underscore the divergent roles of immune responses to different pathogens in the pathogenesis of SLE.^[29]^ In MS, the role of EBV EBNA-1 antibodies presents a starkly opposite pattern to that observed in SLE. GSMR analysis indicated that elevated EBNA-1 antibody levels were significantly associated with an increased risk of MS (OR = 1.266, *P* = 7.98 × E–04, FDR = 1.80 × E–02), a result further corroborated by 2-sample MR analysis (OR = 1.650, *P* = 8.00 × E–03). This discrepancy might be attributed to the distinct pathological characteristics of MS and SLE. MS is characterized by central nervous system (CNS) demyelination, driven primarily by immune-mediated neuroinflammation.^[30,31]^ EBNA-1 antibodies might contribute to MS pathogenesis through molecular mimicry, whereby cross-reactivity between pathogen antigens and host myelin proteins leads to autoimmune attacks on myelin.^[32,33]^ This phenomenon suggests that EBNA-1 antibodies could inadvertently prime the immune system against CNS components. Moreover, persistent EBV infection may exacerbate CNS inflammation by activating microglial cells and intensifying neural damage.^[34]^ Notably, HHV-7 U14 antibodies were associated with increased risk in both SLE and MS, although the underlying mechanisms might vary according to the target tissue. In SLE, HHV-7 could contribute to systemic inflammatory responses, disrupting immune regulation across multiple organs. In MS, HHV-7 may play a more localized role by exacerbating CNS inflammation and immune activation.^[35,36]^ HHV-7’s U14 protein has been shown to facilitate immune evasion by suppressing antiviral host responses, potentially fostering a pro-inflammatory environment conducive to disease progression. The findings highlight that antibody-mediated immune responses may confer either protective effects or heightened risks depending on the pathogen involved, the host’s genetic background, and the immune microenvironment of the target tissue. For instance, EBV-associated antibodies appear protective in SLE but are linked to increased risk in MS. This dichotomy may reflect the differential impact of EBV latent infection on peripheral immune systems versus CNS-specific immune environments. Moreover, pathogen antibody levels likely represent 1 facet of disease risk rather than a singular causal factor. Factors such as the host’s immune regulation capacity, T-cell and B-cell activation status, and the interplay of environmental and genetic influences collectively shape the impact of antibody levels on disease susceptibility.

The reverse causal effects of autoimmune diseases on antibody-mediated immune responses can be explained by several potential mechanisms. Autoimmune diseases, such as SLE and MS, are characterized by systemic or localized immune hyperactivation, which may result in exaggerated antibody responses to exogenous pathogens, as evidenced by elevated antibody levels against specific viruses like varicella-zoster virus and EBV. Molecular mimicry between host autoantigens and pathogen antigens may also contribute, with pathogen-specific antibodies potentially misrecognizing self-tissues and perpetuating autoimmune processes. Dysregulated B-cell function, including the proliferation of autoreactive B cells and overproduction of antibodies, further enhances humoral immune responses. Chronic viral infections, such as EBV, may act as environmental triggers of autoimmune diseases while simultaneously inducing robust immune responses against viral antigens. Additionally, persistent inflammation and increased antigen presentation associated with autoimmune diseases may amplify antibody production against viral antigens. Together, these mechanisms elucidate the observed bidirectional interactions, whereby autoimmune diseases influence antibody-mediated immune responses, highlighting the intricate interplay between autoimmunity and pathogen-specific immunity and offering valuable insights for therapeutic strategies.

This study, while comprehensive, is not without limitations. The reliance on MR assumes that all genetic variants used as instruments affect the risk of autoimmune diseases only through their impact on antibody levels, a condition known as the exclusion-restriction criterion. However, pleiotropy, where genetic variants influence multiple traits, could confound these results. Additionally, the interpretation of our findings is limited to the populations from which the genetic data were derived, predominantly of European descent. Future research should focus on longitudinal studies to better understand the temporal dynamics between viral antibody levels and the development of autoimmune diseases. Experimental studies could also explore the immunological mechanisms underlying these associations in vivo. Furthermore, investigating other potential viral or environmental factors that contribute to the pathogenesis of autoimmune diseases could provide a more comprehensive understanding of these complex conditions.

## 5. Conclusion

Bidirectional MR analysis revealed protective effects of EBV EBNA-1 and ZEBRA antibodies on SLE, while HHV-7 U14 antibodies increased SLE risk. Conversely, EBV EBNA-1 antibodies elevated MS risk. A bidirectional causal relationship was observed between SLE and MS, offering new insights into disease mechanisms and potential therapies. Limitations include population restrictiveness and genetic pleiotropy, requiring further validation.

## Acknowledgments

We extend our heartfelt gratitude for the wealth of resources provided by the public database, which has offered invaluable support to our work and research.

## Author contributions

**Formal analysis:** Luofei Huang.

**Investigation:** Luofei Huang.

**Software:** Li Han, Quanzhi Lin.

**Validation:** Quanzhi Lin.

**Writing – original draft:** Luofei Huang.

**Writing – review & editing:** Li Han, Quanzhi Lin.

## Supplementary Material




