# Supplementary figures and images for: Bidirectional causal relationships between antibody-mediated immune responses and autoimmune diseases: Insights from Mendelian randomization analysis
Source: Medicine (Baltimore). 2026 Jan 2;105(1):e47013. doi: 10.1097/MD.0000000000047013 (PMC12778236; doi:10.1097/MD.0000000000047013)

A

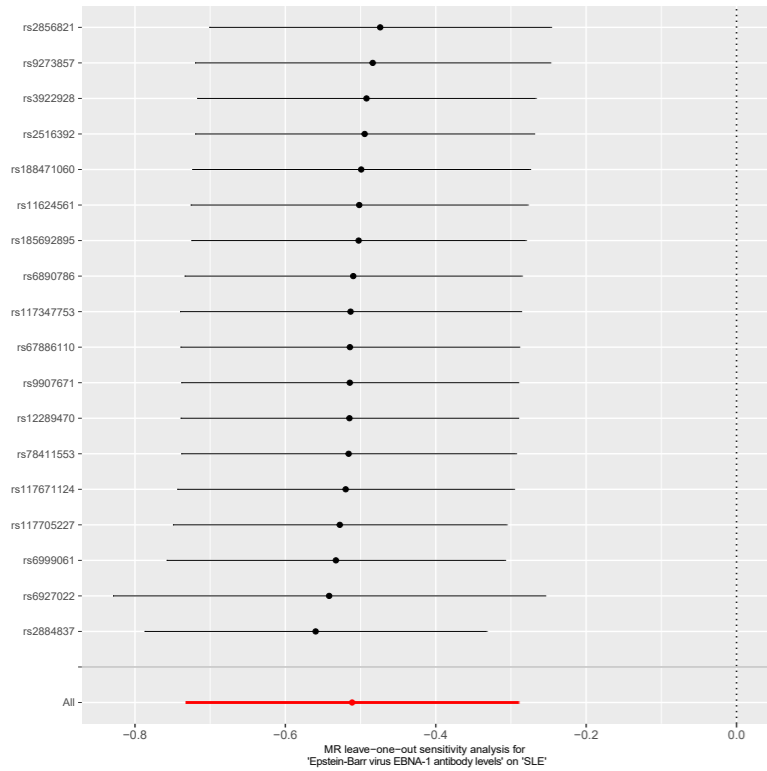

B

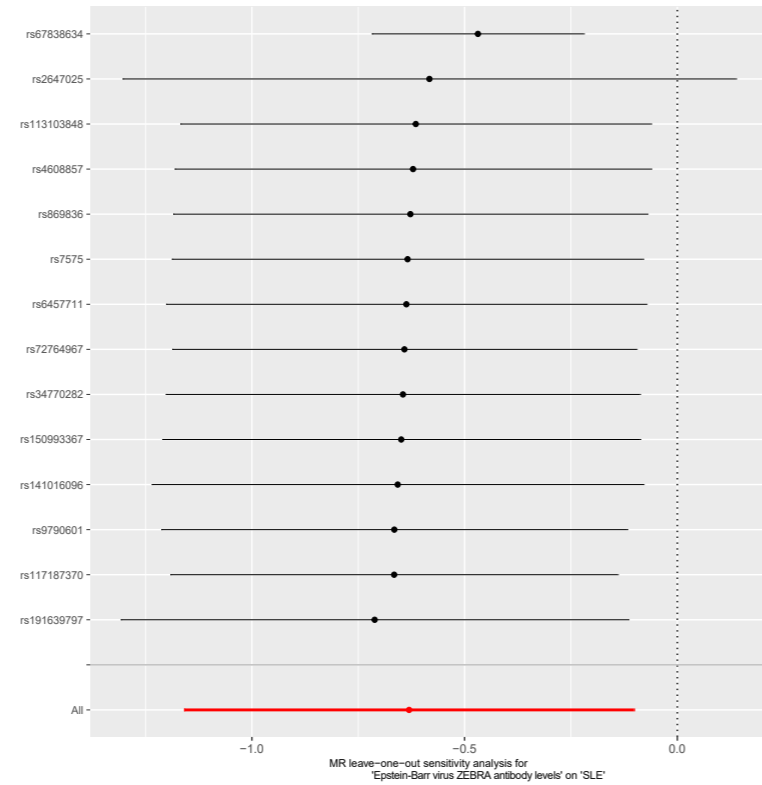

C

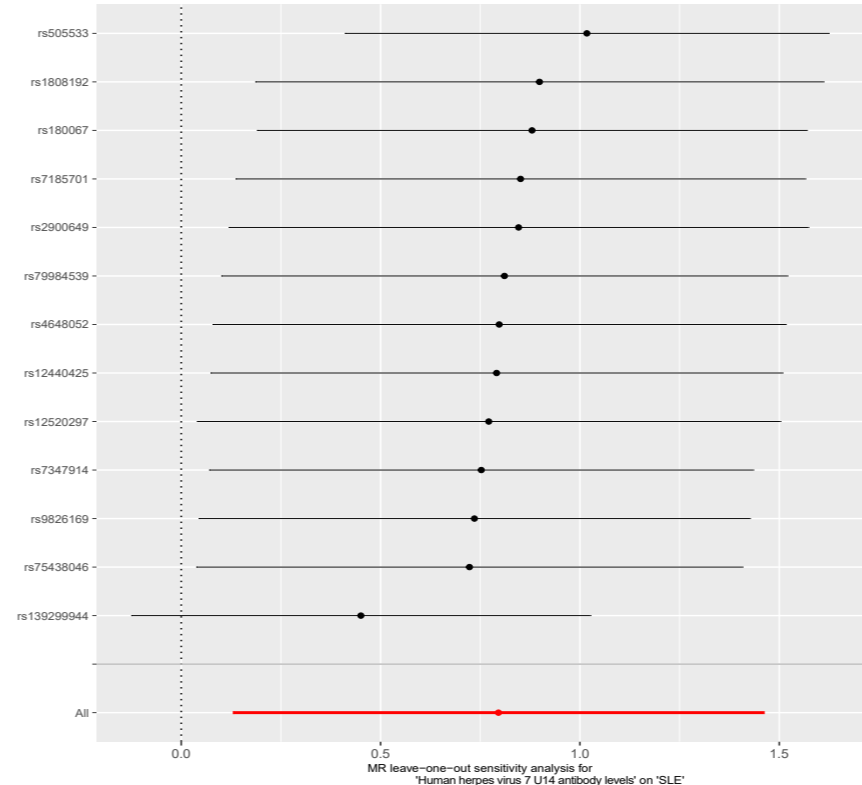

D

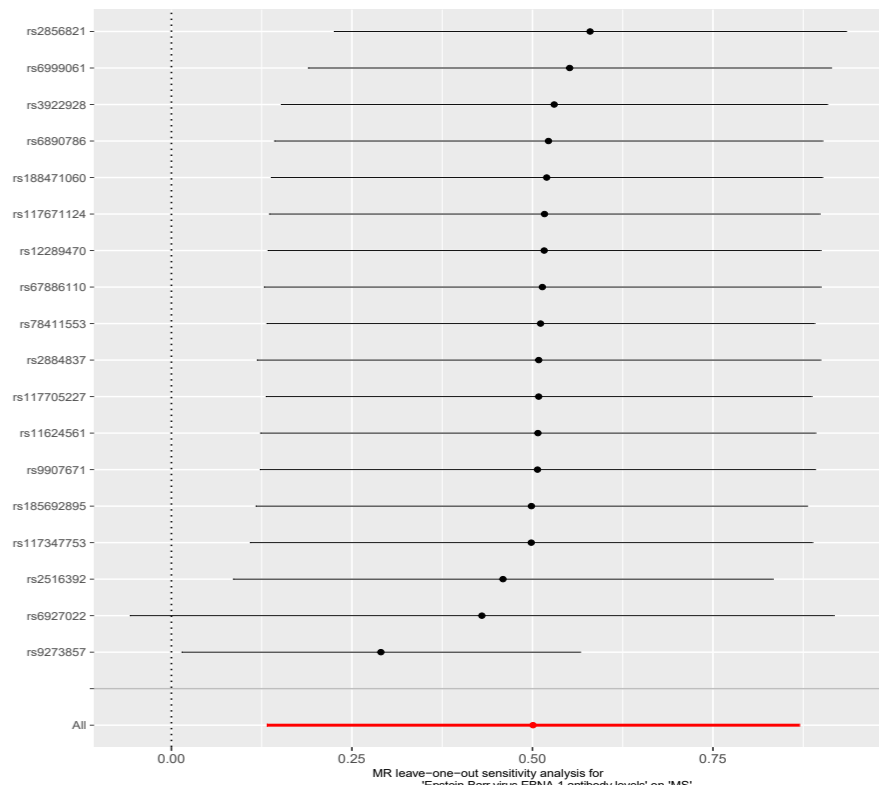

E

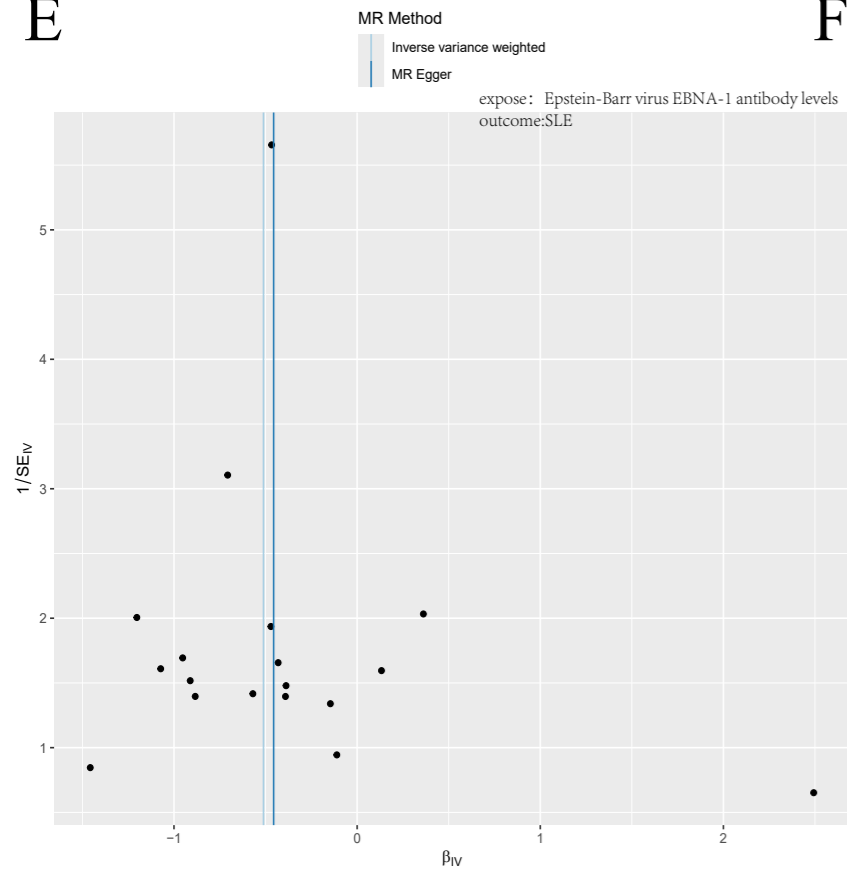

F

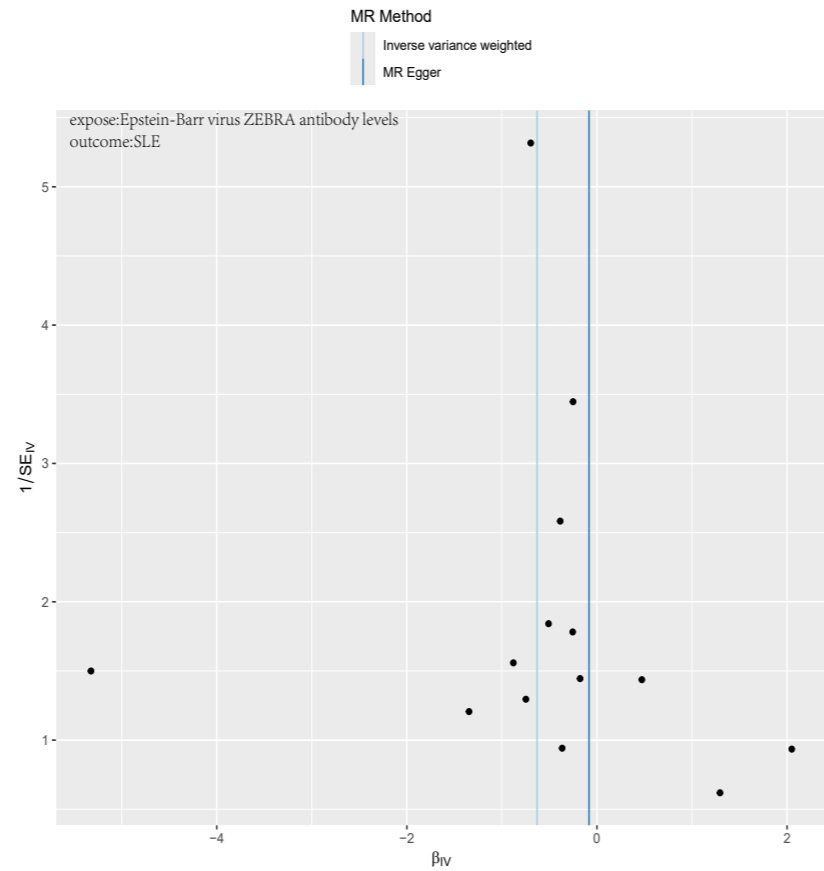

G

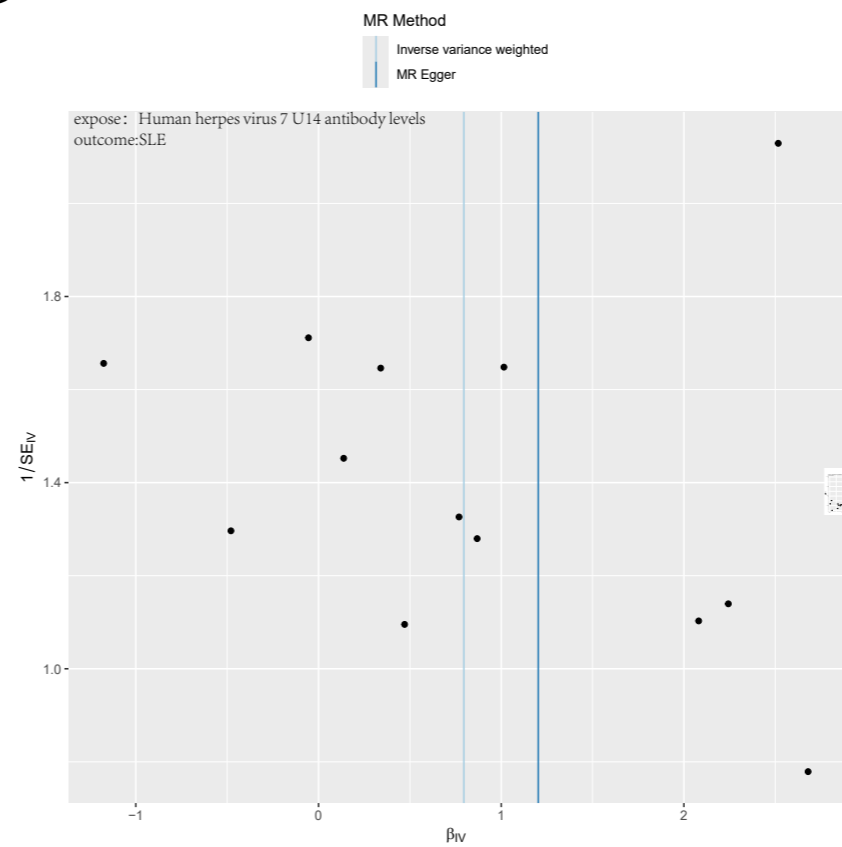

H

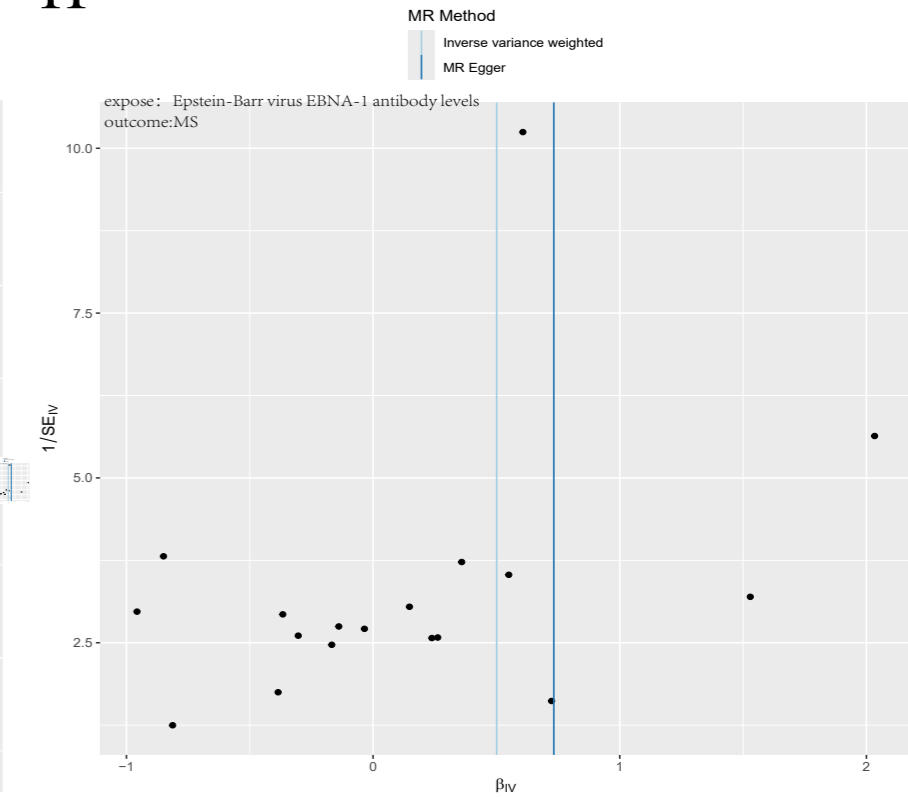

A

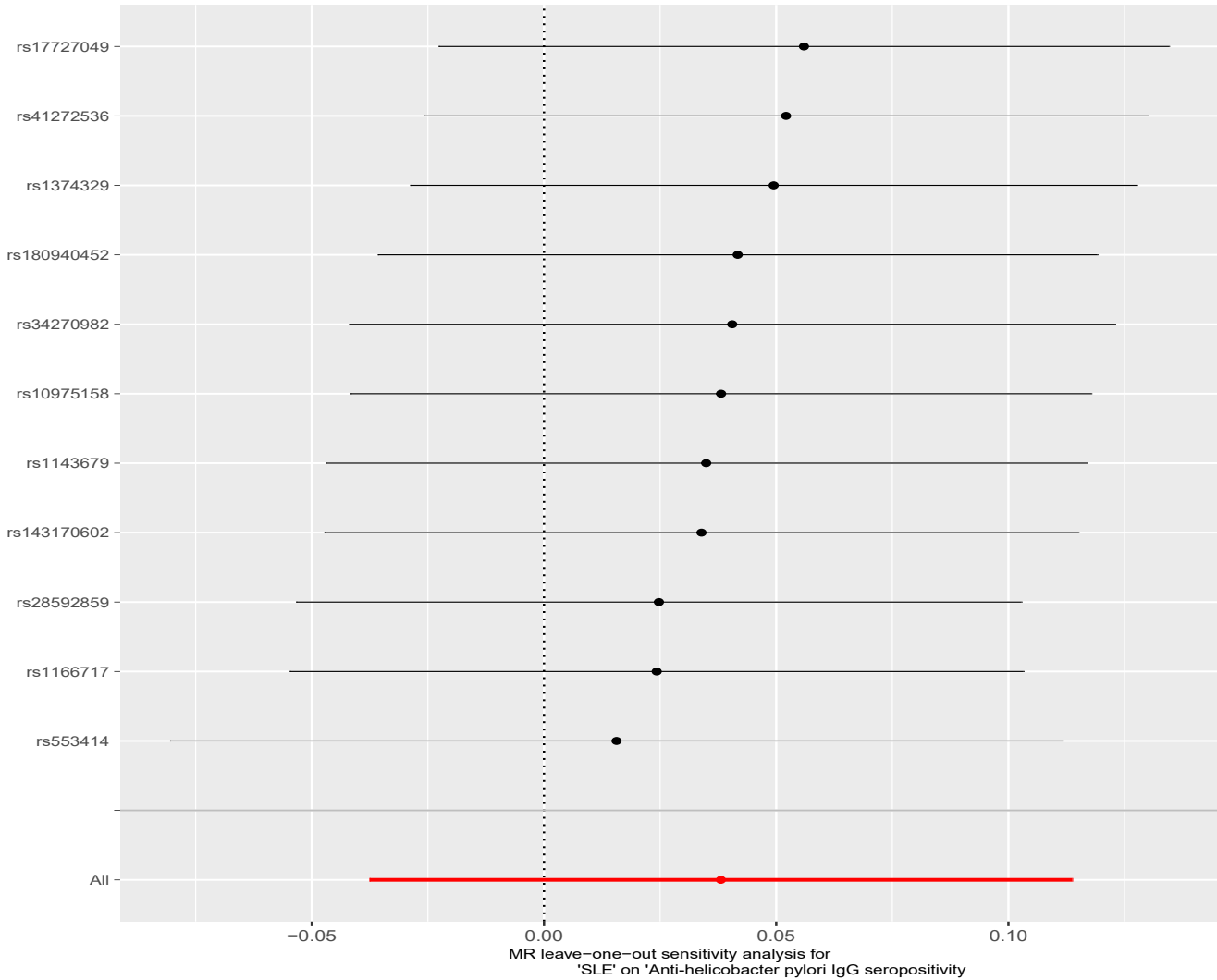

B

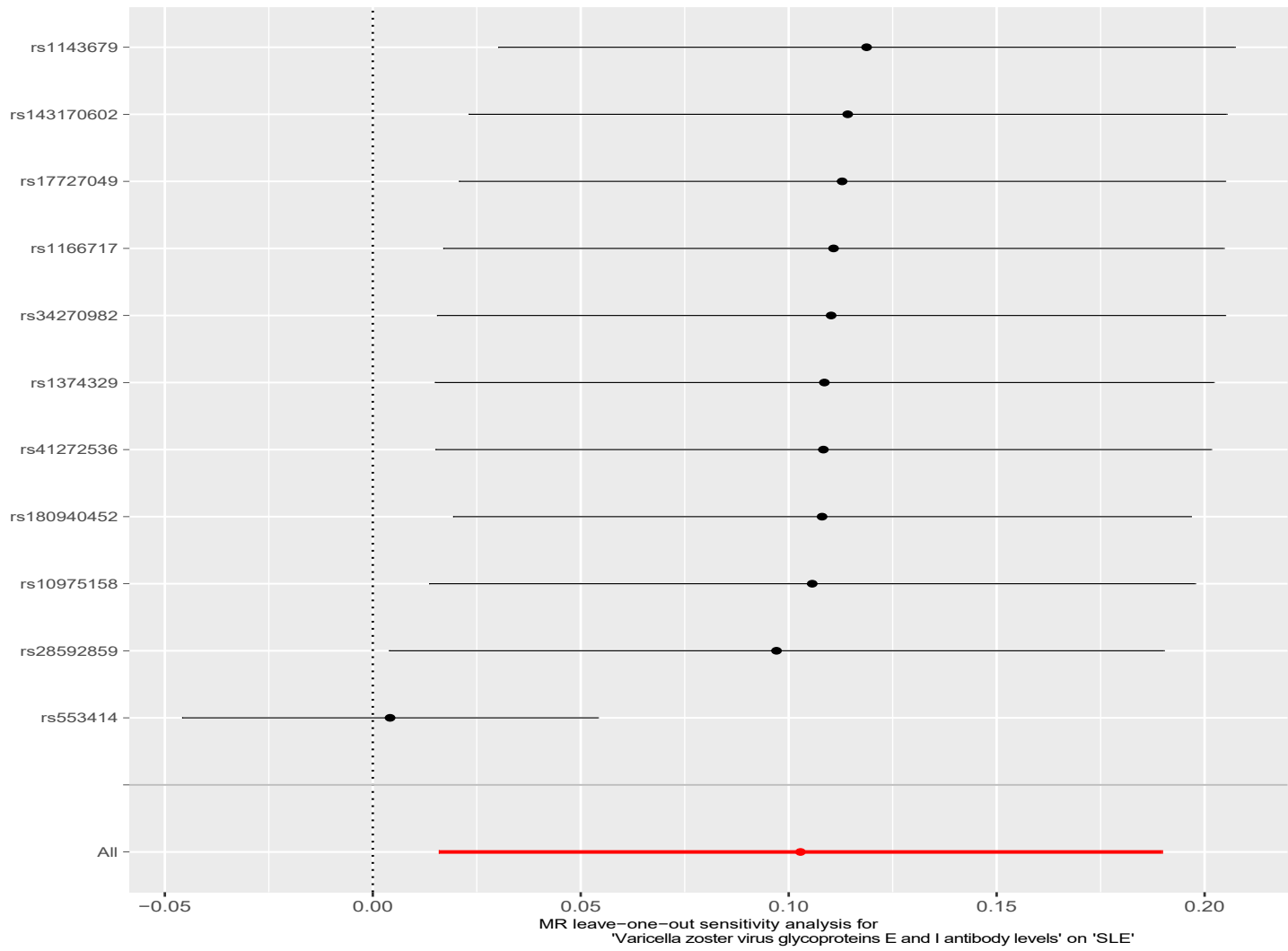

C

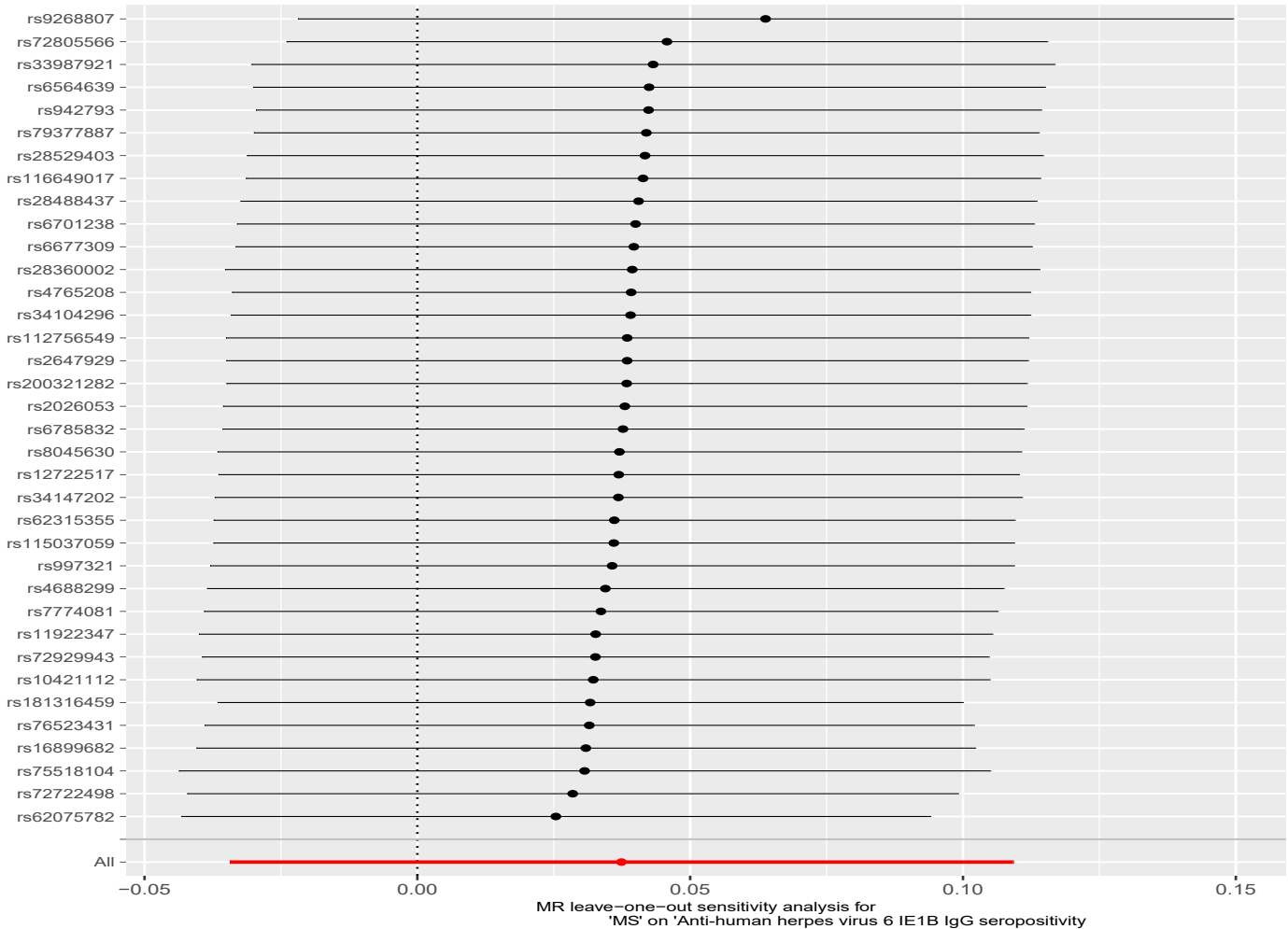

D

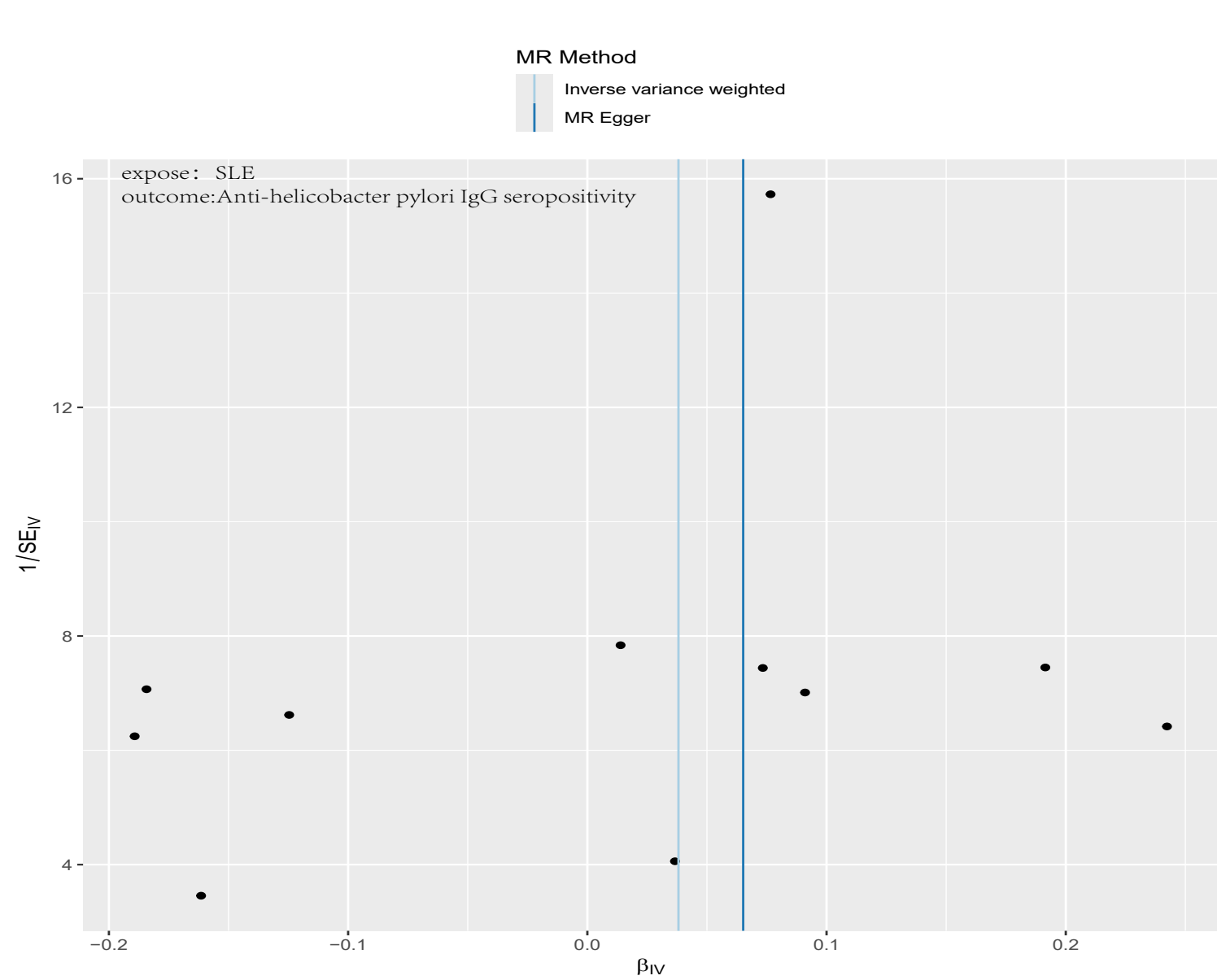

E

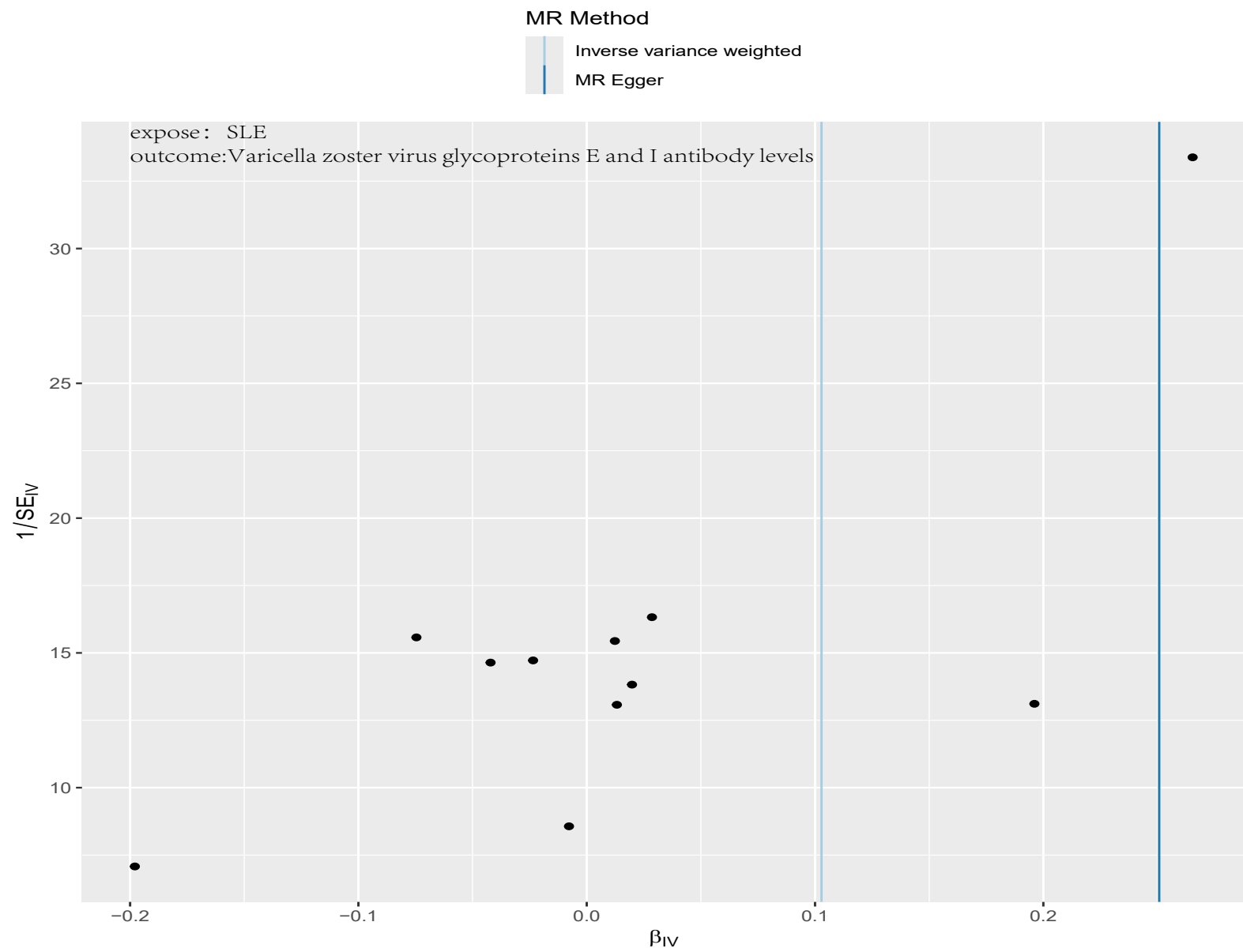

F

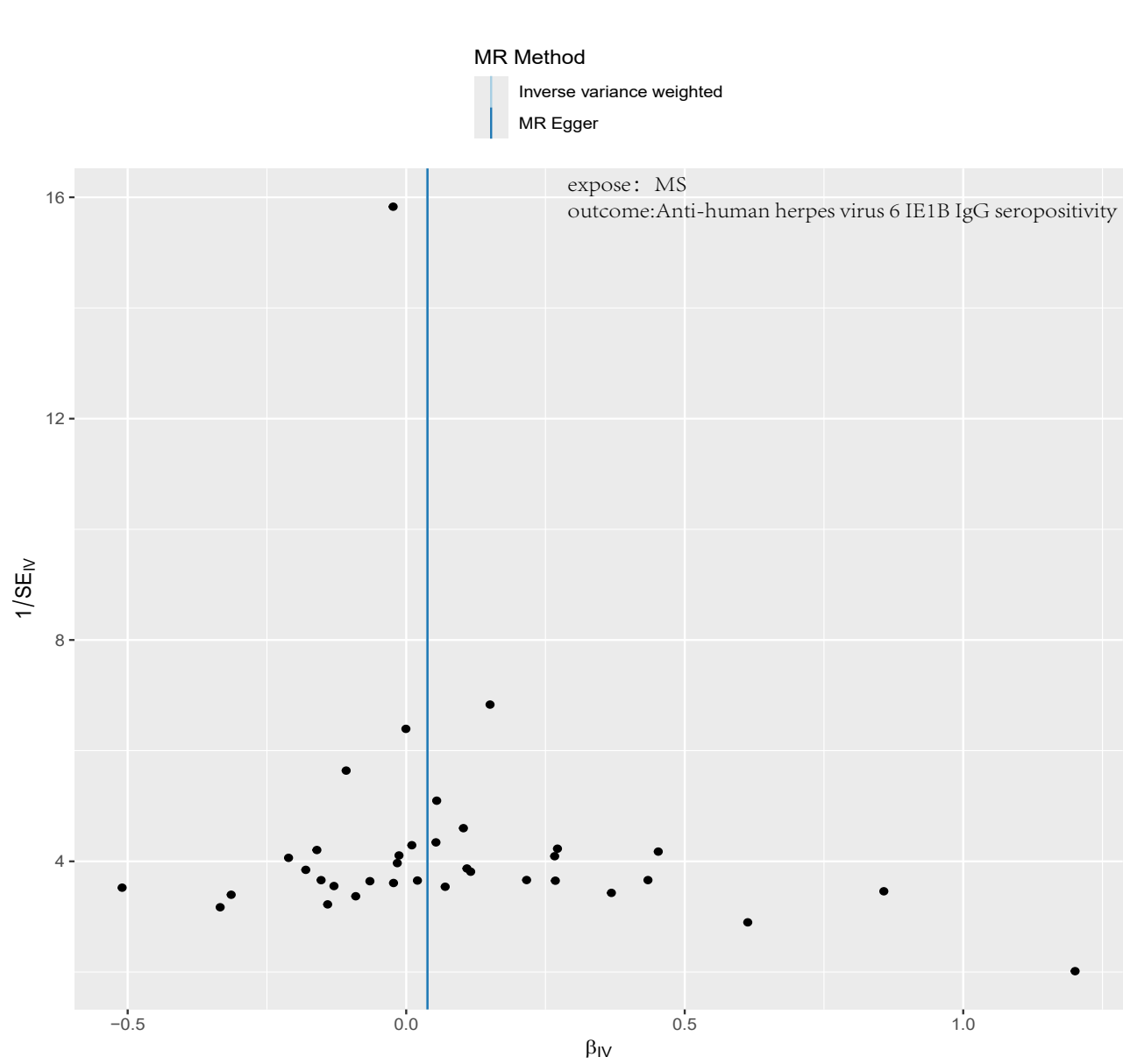

Supplement: Supplementary file 2 [file medi-105-e47013-s002.pdf]
